# Diverse Manifestations of Central Nervous System Tuberculosis: Magnetic Resonance Imaging (MRI) Presentations and Laboratory Investigations in a Cohort Study

**DOI:** 10.7759/cureus.87077

**Published:** 2025-06-30

**Authors:** Tarang Patel, Virendra K Meena, Ashwani K Khandekar, Kesha Rachani, Pinki Meena, Deepa Shukla

**Affiliations:** 1 Pathology, All India Institute of Medical Sciences (AIIMS) Rajkot, Rajkot, IND; 2 Radiodiagnosis, Geetanjali Medical College & Hospital, Udaipur, IND; 3 Anaesthesiology, Geetanjali Medical College & Hospital, Udaipur, IND; 4 Yoga and Naturopathy, All India Institute of Medical Sciences, Jodhpur, Jodhpur, IND

**Keywords:** antitubercular therapy, central nervous system tuberculosis, magnetic resonance imaging, meningitis, neuroimaging, tuberculoma

## Abstract

Objectives: This study aims to evaluate and characterize the diverse MRI findings in central nervous system (CNS) tuberculosis (TB) and the role of other laboratory investigations to aid in early detection and appropriate management.

Materials and methods: This retrospective, cross-sectional study analyzed clinical and imaging data from 43 patients with confirmed CNS TB. The diagnosis was confirmed through cerebrospinal fluid (CSF) analysis, biopsy, or clinical and radiological improvement post-antitubercular therapy (ATT). MRI findings were categorized into meningeal and parenchymal forms, with further subtyping based on lesion characteristics. Chi-square statistics were performed using IBM SPSS Statistics for Windows, version 27 (IBM Corp., Armonk, New York, United States) to correlate CNS TB with clinical parameters.

Results: The mean age of the 43 patients was 33 years, and 58% were male. The most common clinical symptoms were headache (86%) and fever (79%). MRI findings revealed meningeal TB (leptomeningitis and pachymeningitis) and parenchymal TB (tuberculomas, tubercular abscesses, cerebritis, rhombencephalitis, and encephalopathy). Frequent observations included ring-enhancing lesions and perilesional edema. Parenchymal tuberculomas showed varying stages. Advanced imaging techniques such as magnetic resonance spectroscopy and perfusion imaging were useful in differentiating tuberculomas from neoplastic and infectious differentials.

Conclusion: CNS TB presents with diverse MRI patterns, including both typical and atypical manifestations. Accurate radiological assessment, combined with clinical correlation, is essential for early diagnosis and management. Prompt initiation of ATT is critical in preventing long-term neurological complications. Future research should focus on refining imaging biomarkers to improve diagnostic accuracy.

## Introduction

Tuberculosis (TB), caused by *Mycobacterium tuberculosis*, is responsible for over eight million deaths globally due to its direct or indirect effects [1]. The resurgence of TB, particularly in non-endemic regions, has been linked to the rise of acquired immunodeficiency syndrome (AIDS), with 9,272 cases reported in the United States in 2016 [2]. Central nervous system (CNS) involvement represents the most severe form of TB, significantly contributing to morbidity and mortality, often leaving permanent sequelae in affected individuals [3,4].

CNS TB includes 10% of all site TB cases and 20% of TB cases in immunocompromised patients [5]. *M. tuberculosis* is nearly always the causative agent, typically spreading to the CNS via the hematogenous route, often from a pulmonary source. However, rare cases of direct spread from the orbit, mastoids, or paranasal sinuses have been reported [6]. Once in the CNS, the bacterium elicits a strong granulomatous inflammatory response, which is usually detectable on magnetic resonance imaging (MRI), facilitating radiological diagnosis and timely treatment. Cranial TB commonly presents as focal tuberculoma, hydrocephalus, or tubercular meningitis (TBM) on MRI. However, a small subset of cases poses diagnostic challenges due to atypical imaging findings, potentially leading to delayed or incorrect diagnoses. This study seeks to evaluate and describe the range of typical and atypical MRI findings associated with cranial TB.

## Materials and methods

This was a retrospective study conducted in a tertiary care hospital, Geetanjali Medical College & Hospital, Udaipur, Rajasthan, India, involving the analysis of clinical records, laboratory investigations, and imaging studies for patients diagnosed with CNS TB over five years from January 2020 through December 2024. The study was approved by the Human Research Ethics Committee, Geetanjali University (approval number: GU/HREC/EC/2025/2683).

Inclusion and exclusion criteria

Patients whose diagnosis of CNS TB was confirmed by CSF findings with at least one of the following positive test results: CSF cytology, CSF culture, CSF ADA, and tissue biopsy results consistent with tubercular etiology, and who showed clinical and radiological improvement after antitubercular therapy (ATT), were included in the study. Patients who lacked comprehensive radiological investigations were excluded from the study.

Methodology

By reviewing the patient's medical records, a comprehensive medical history and the findings of the physical examination were assembled. MRI was performed using the SIGNA™ Architect 3 Tesla MRI system (GE Healthcare Technologies, Inc., Chicago, Illinois, United States). Axial precontrast T1-weighted (T1W), T2-weighted (T2W), fluid-attenuated inversion recovery sequence (FLAIR), diffusion-weighted imaging (DWI), apparent diffusion coefficient (ADC), gradient echo sequence(GRE)/susceptibility-weighted imaging (SWI), and postcontrast T1W and FLAIR sequences were done for suspected intracranial tuberculosis. When the appearance or distribution of the lesions was abnormal and posed a diagnostic challenge, magnetic resonance spectroscopy (MRS) may also have been used. When vascular complications were suspected, magnetic resonance angiography (MRA) was done. The radiological findings were reviewed by expert radiologists. Characteristics of contrast enhancement and signal intensity were evaluated. Signal intensity was compared with gray matter for brain imaging on the T1WI and T2WI MRI signals.

Statistical analysis

Statistical analysis employed descriptive statistical methods. The level of significance was set at 5%. The chi-square test of independence was performed using IBM SPSS Statistics for Windows, version 27 (IBM Corp., Armonk, New York, United States) to analyze its association with the incidence of seizure and extracranial TB incidence.

## Results

Demographic and clinical findings

A total of 43 cases of cranial tuberculosis were identified in the given period. The age varied from 12 to 56 years, with a mean age of 33 years. The male and female patients were 25 (58%) and 18 (42%), respectively, with a slight male predominance. The data regarding clinical presentation are described in Table 1. The most common clinical symptoms were headache and fever, seen in 37 (86%) and 34 (79%) cases, respectively. Extracranial involvement was in the spine (n=10, 23.2%), lung/pleura (n=9, 20.9%), and abdomen (n=4, 9.3%). Associated viral infection was present in 11 (25.58%) cases. Comorbidity was present in 18 (41.8%) cases (Table 1).

**Table 1:** Clinical data of the patients (N=43)

Presentation	Frequency (Percentage)
Fever	34 (79.06%)
Weight loss	10 (23.25%)
Headche	37 (86.04%)
Loss of appetite	25 (58.13%)
Paresis	16 (37.20%)
Extracranial : Spine involvement	10 (23.25%)
Extracranial : Lung/Pleural involvement	9 (20.93%)
Extracranial : Abdominal involvemen	4 (9.30%)
Associated viral infection	11 (25.58%)
Associated comorbidity : Diabetes or Hypertension	18 (41.86%)

Laboratory and imaging findings

**Figure 1:**
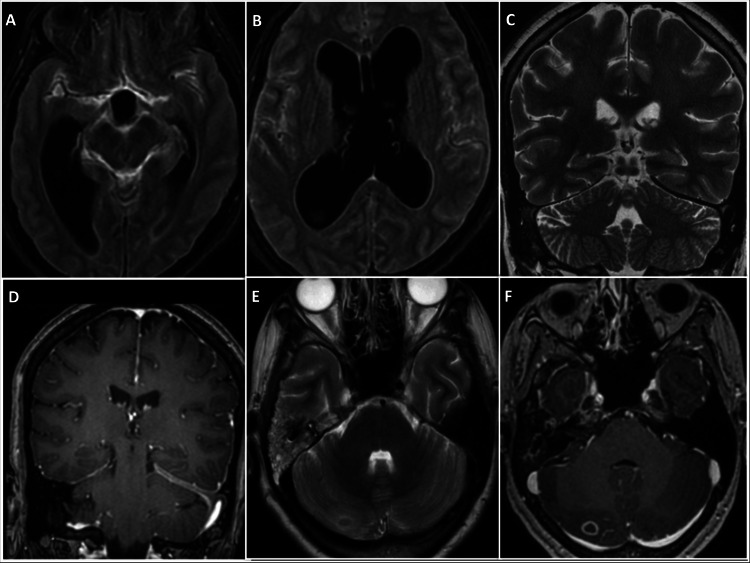
Imaging features in leptomeningitis, pachymeningitis, and tuberculoma (A, B) Leptomengitis with hydrocephalous: Post-contrast axial fluid-attenuated inversion recovery sequence (FLAIR) image demonstrates leptomeningitis affecting the bilateral Sylvian fissures, suprasellar cistern, and sulcal spaces, accompanied by dilation of the bilateral lateral and third ventricles. (C, D) Pachymeningitis : Coronal T2-weighted image shows hypointense thickening of the left-side tentorium (C). Coronal post-contrast T1-weighted image demonstrates dural enhancement along the left-side tentorium (D). (E, F) Tuberculoma: Axial T2-weighted image reveals a T2 hypointense lesion with mild perilesional edema (E). Post-contrast T1-weighted image demonstrates peripheral enhancement around the lesion (F).

**Figure 2:**
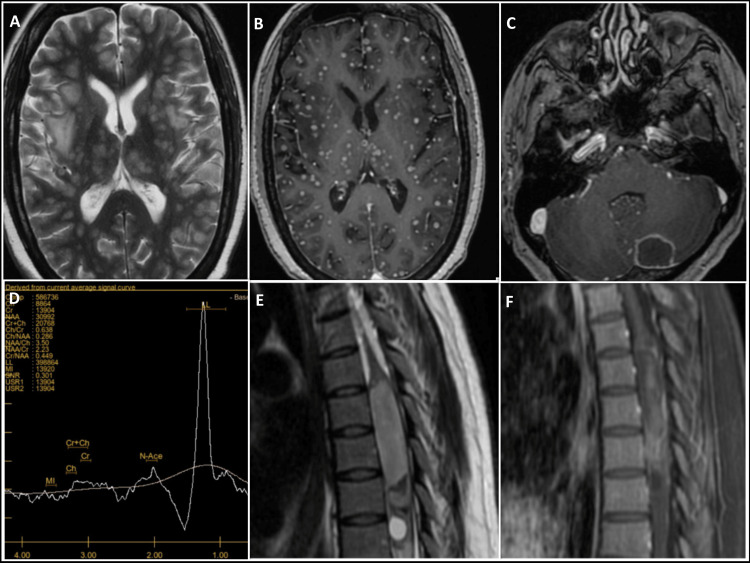
Imaging features in miliary tuberculoma, pachymeningitis, and tuberculosis abscess (A, B) Miliary Tuberculoma: Numerous  lesions scattered throughout the bilateral cerebral hemispheres and basal ganglia, accompanied by surrounding edema (A). Nodular enhancement of lesions in the bilateral cerebral hemispheres and basal ganglia (B). (C, D) Pachymeningitis: Axial T2-weighted image reveals a hypointense lesion in the left cerebellum, accompanied by mild perilesional edema and peripheral ring enhancement (C). Magnetic resonance spectroscopy of the lesion demonstrates a lipid-lactate peak along with a reduced NAA peak (D). (E, F) Tuberculosis Abscess: Sagittal T2-weighted image of the dorsal spinal cord shows a heterogeneously hyperintense lesion (E). Sagittal post-contrast T1-weighted fat-saturation image reveals peripheral enhancement along with leptomeningeal enhancement in the surrounding area (F).

**Figure 3:**
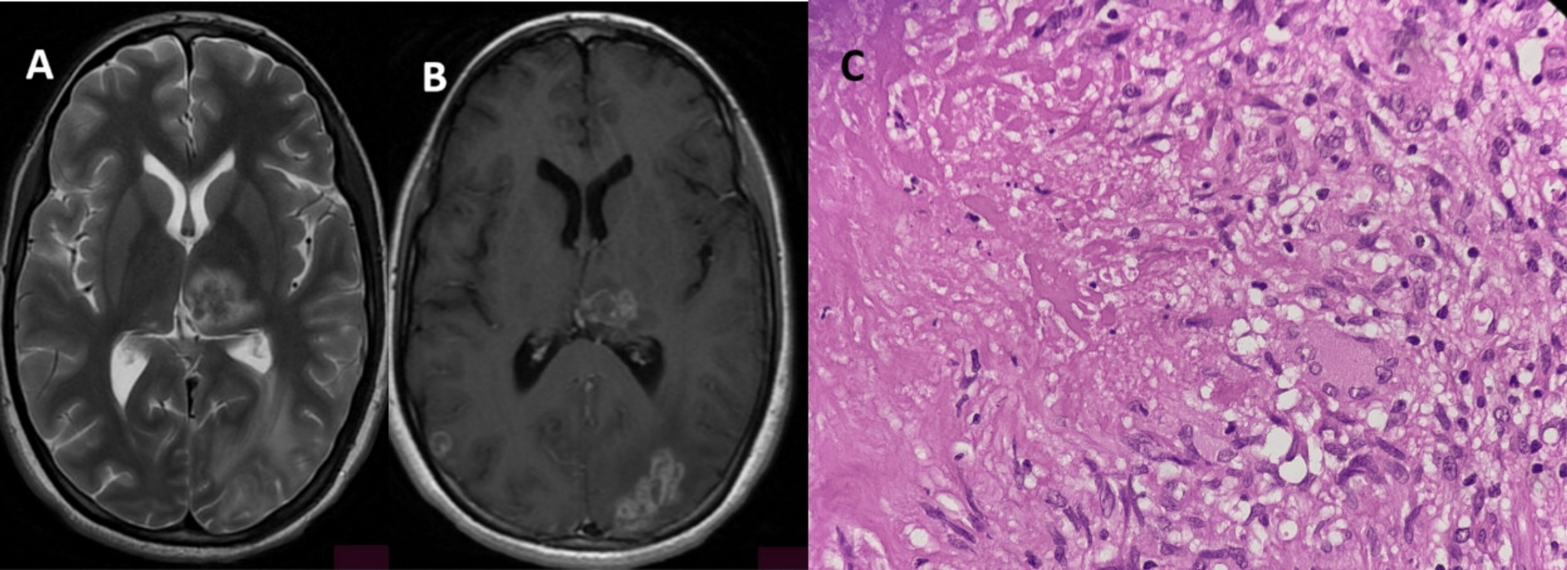
Imaging and histopathological features of mass-like tuberculosis (A) Axial T2-weighted image demonstrates hypointense lesions in the left thalamus and left occipital region with surrounding perilesional edema; (B) Post-contrast axial T1-weighted image reveals a conglomerate ring-enhancing lesion in the left thalamus and left occipital region, along with an additional ring-enhancing lesion in the right temporal region; (C) Histopathology shows caseating necrosis surrounded by palisading epithelioid histiocytes mixed with few lymphocytes and Langhans giant cells (H&E, 40x).

The list of laboratory investigations and MRI methods performed to assist in TB diagnosis is given in Table 2. Regarding laboratory investigations, CSF cytology was the most frequently performed test, utilized in 88% of cases (38 out of 43), reflecting its fundamental role in CSF analysis. CSF ADA and CSF culture were also commonly employed, used in 70% and 63% of cases, respectively, indicating their importance in detecting tubercular pathology. CSF polymerase chain reaction (PCR) and tissue biopsy were performed in 40% (17 out of 43) of the cases each, likely reserved for complex cases or those with atypical presentations where higher diagnostic precision was needed. Among the radiological diagnoses, "leptomeningitis and tuberculoma” emerged as the most common, seen in 15 cases (35%), indicating frequent coexistence of meningeal and parenchymal tuberculosis in the central nervous system (CNS). This was followed by isolated leptomeningitis in six cases and tuberculoma in four cases. Other less frequent findings included pachymeningitis (three cases), tumor-like lesions, tubercular abscess, tubercular cerebritis, and rhombencephalitis, often in combination with leptomeningitis (Table 2), (Figures 1-3). 

**Table 2:** Laboratory investigations performed and radiological diagnosis (N=43) *percentages are calculated on the total of 43 cases. CSF: cerebrospinal fluid; ADA: adenosine deaminase; PCR: polymerase chain reaction

Investigations Performed	Radiological Findings	Frequency (Percentage*)
CSF cytology and CSF ADA	Tuberculoma	1 (2.3%)
CSF cytology, CSF culture and CSF ADA	Tuberculoma	1 (2.3%)
CSF culture, CSF ADA, CSF PCR	Tuberculoma	1 (2.3%)
CSF cytology, CSF culture, CSF ADA, tissue biopsy	Tuberculoma	1 (2.3%)
CSF cytology, CSF culture, CSF ADA, tissue biopsy, CSF PCR	Giant tuberculoma	1 (2.3%)
CSF cytology, CSF culture, CSF ADA, tissue biopsy	Tubercular abscess	1 (2.3%)
CSF cytology, CSF culture, CSF ADA, tissue biopsy	Tubercular cerebritis	1 (2.3%)
CSF cytology, CSF culture, CSF ADA	Tuberculous encephalopathy	1 (2.3%)
CSF cytology and tissue biopsy	Tumor-like appearance lesion	1 (2.3%)
CSF cytology, tissue biopsy, CSF PCR	Tumor-like appearance lesion	1 (2.3%)
CSF cytology, CSF culture, CSF ADA	Leptomeningitis	2 (4.7%)
CSF cytology, CSF culture, CSF ADA, tissue biopsy	Leptomeningitis	2 (4.7%)
CSF cytology, CSF culture, CSF ADA, CSF PCR	Leptomeningitis	1 (2.3%)
CSF culture, CSF ADA, CSF PCR, tissue biopsy	Leptomeningitis	1 (2.3%)
CSF cytology and CSF ADA, CSF PCR	Pachymeningitis	2 (4.7%)
CSF cytology, CSF culture, CSF ADA, tissue biopsy	Pachymeningitis	1 (2.3%)
CSF ADA and tissue biopsy, CSF PCR	Pachymeningitis	1 (2.3%)
CSF cytology and CSF PCR	Leptomeningitis and tuberculoma	3 (7.0%)
CSF ADA and tissue biopsy	Leptomeningitis and tuberculoma	1 (2.3%)
CSF cytology and CSF ADA, CSF PCR	Leptomeningitis and tuberculoma	2 (4.7%)
CSF cytology, CSF ADA and tissue biopsy	Leptomeningitis and tuberculoma	1 (2.3%)
CSF cytology and CSF culture, CSF PCR	Leptomeningitis and tuberculoma	2 (4.7%)
CSF cytology, CSF culture, and CSF ADA	Leptomeningitis and tuberculoma	4 (9.3%)
CSF cytology, CSF culture, CSF ADA, tissue biopsy	Leptomeningitis and tuberculoma	1 (2.3%)
CSF cytology and tissue biopsy	Leptomeningitis and giant tuberculoma	1 (2.3%)
CSF cytology and tissue biopsy	Leptomeningitis and tubercular abscess	1 (2.3%)
CSF cytology and CSF culture, CSF PCR	Leptomeningitis and tubercular abscess	1 (2.3%)
CSF cytology, CSF ADA and CSF culture	Leptomeningitis and tubercular abscess	1 (2.3%)
CSF cytology and CSF culture	Leptomeningitis and tubercular abscess	1 (2.3%)
CSF cytology and CSF culture	Leptomeningitis and tubercular cerebritis	1 (2.3%)
CSF ADA and tissue biopsy, CSF PCR	Leptomeningitis and tubercular cerebritis	1 (2.3%)
CSF cytology and CSF culture	Leptomeningitis and tuberculous rhombencephalitis	1 (2.3%)
CSF cytology, CSF ADA, CSF PCR	Leptomeningitis and tuberculous rhombencephalitis	1 (2.3%)

TB on MRI was classified into meningeal, parenchymal, and mixed types. Parenchymal TB included six subtypes: tuberculomas (four cases, isointense to hypointense on T1W, hypointense on T2W, no diffusion restriction, lipid-lactate peak on MRS, ring enhancement, perilesional edema), giant tuberculoma (one case, similar features with mass effect), tubercular abscess (one case, T2W hyperintense, diffusion restriction), tubercular cerebritis (one case, T1W hypointense to isointense, T2W hyperintense, possible diffusion restriction, gyriform enhancement), tuberculous encephalopathy (one case, diffuse T2 hyperintensity, white matter enhancement), and tumor-like lesions (two cases, T1W isointense, T2W hypointense, occasional lipid-lactate peaks, intense enhancement, edema). Meningeal TB included leptomeningitis (six cases) and pachymeningitis (three cases), both showing meningeal contrast enhancement without distinct T1W/T2W changes. Mixed type included five subtypes: leptomeningitis with tuberculoma (15 cases, tuberculoma features plus leptomeningeal enhancement, often with hydrocephalus, ventriculitis, vasculitis), leptomeningitis with giant tuberculoma (one case, similar with larger size, mass effect), leptomeningitis with tubercular abscess (four cases, T1W hypointense, T2W hyperintense, diffusion restriction, lipid-lactate peaks, ring enhancement, meningeal inflammation), leptomeningitis with tubercular cerebritis (two cases, cerebritis features), and leptomeningitis with tuberculous rhombencephalitis (two cases, T1W hypointense, T2W hyperintense, basal cisternal enhancement, brainstem involvement) (Table 3).

**Table 3:** MRI features according to type of tuberculosis T1W: T1-weighted; T2W: T2-weighted; DWI: diffusion-weighted imaging; MRS: magnetic resonance spectroscopy; T1WCE: T1-weighted contrast-enhanced

Type of Tuberculosis	Cases, n (%)	T1W	T2W	DWI	MRS	T1WCE	Additional findings
Parenchymal							
Tuberculoma	4 (9.30%)	Isointense to hypointense	Hypointense	No restriction	Lipid lactate peak	Ring enhancement	Perilesional edema
Giant tuberculoma	1 (2.33%)	Isointense	Hypointense	No restriction	Lipid lactate peak	Ring enhancement	Perilesional edema
Tubercular abscess	1 (2.33%)	Hypointense	Hyperintense	Diffusion restriction	Lipid lactate peak	Ring enhancement	Perilesional edema
Tubercular cerebritis	1 (2.33%)	Hypointense to Isointense	Hyperintense	Diffusion restriction may be present	_	Gyriform enhancement	_
Tuberculous encephalopathy	1 (2.33%)	_	Hyerintense due to cerebral edema	_	_	Enhancement seen in involved white matter.	_
Tumor-like appearance	2 (4.65%)	Isointense	Hypointense	Usually not seen	Lipid lactate may be seen	Intense contrast enhancement	Perilesional edema
Meningeal							
Lepto-meningitis	6 (13.95%)	_	_	_	_	Lepto-meningeal enhancement	
Pachymeningitis	3 (6.98%)	_	_	_	_	Pachy-meningeal enhancement	
Mixed							
Lepto-meningitis and tuberculoma	15 (34.88%)	Tuberculoma appears isointense to hypointense	Tuberculoma appears hypointense	No restriction	Lipid lactate peak in tuberculoma	Lepto-meningeal enhancement in lepto-meningitis and ring enhancement in tuberculoma	Hydro-cephalus, ventriculitis and arterial vasculitis
Lepto-meningitis and giant tuberculoma	1 (2.33%)	Giant tuberculoma appears isointense to hypointense	Giant tuberculoma appears hypointense	No restriction	Lipid lactate peak in giant tuberculoma	Lepto-meningeal enhancement in lepto-meningitis and ring enhancement in giant tuberculoma	Hydro-cephalus and ventriculitis
Lepto-meningitis and tubercular abscess	4 (9.30%)	Tubercular abscess appears hypointense	Tubercular abscess appears hyperintense	Present in tubercular abscess	Lipid lactate peak in TB abscess	Lepto-meningeal enhancement in lepto-meningitis and peripheral enhancement in tubercular abscess	Hydro-cephalus and ventriculitis
Lepto-meningitis and tubercular cerebritis	2 (4.65%)	Tubercular cerebritis appears hypointense to isointense	Tubercular cerebritis appears hyperintense	Diffusion restriction may be present in tubercular cerebritis	_	Lepto-meningeal enhancement in lepto-meningitis and gyriform enhancement in tubercular cerebritis	_
Lepto-meningitis and tuberculous rhombencephalitis	2 (4.65%)	Hypointense	Hyperintense	_	_	Lepto-meningeal enhancement in basal cistern	_

The histopathological analysis of 17 cases of CNS TB in which tissue biopsy was performed revealed a predominance of caseating granulomas, which are a hallmark of TB infection. Of the 17 cases, 12 showed caseating granulomas, and five showed non-caseating granulomas (Table 4).

**Table 4:** Morphology of CNS TB on biopsy according to radiological type (N=17*) *tissue biopsy were performed in 17 of the total 43 cases (40%) TB: tuberculosis; CNS: central nervous system

TB type	Total cases, n	Caseating granuloma, n	Non-caseating granuloma, n
Tuberculoma	2	2	-
Giant tuberculoma	1	1	-
Tubercular abscess	1	1	-
Tumour-like appearance	2	2	-
Leptomeningitis and tuberculoma	8	4	4
Leptomeningitis and giant tuberculoma	1	1	-
Leptomeningitis and tubercular abscess	2	1	1

Statistical analysis

Seizures were most prevalent in parenchymal TB (90%, 9/10) and mixed types (62.5%, 15/24), while rare in purely meningeal TB (11%, 1/9), indicating a strong association between cortical involvement and seizure activity. Similarly, extracranial TB was more commonly observed in parenchymal (70%, 7/10) and mixed cases (54.2%, 13/24) compared to meningeal TB (22%, 2/9), suggesting that systemic dissemination is more frequent in cases with parenchymal or combined CNS involvement. The Chi-square test of Independence shows a significant association between TB type and seizure occurrence (p = 0.002). There is no statistically significant association between TB type and extracranial TB presence (p > 0.05), although parenchymal and mixed forms tend to show more extracranial involvement (Table 5).

**Table 5:** Contingency table of seizure and extracranial location with intracranial TB (N=43) TB: tuberculosis

TB type	Seizure present, n	Seizure absent, n	Extracranial TB present, n	Extracranial TB absent, n
Parenchymal (n=10)	9	1	7	3
Meningeal (n=9)	1	8	2	7
Mixed (n=24)	15	9	13	11

## Discussion

Intracranial TB can be primarily classified into meningeal and parenchymal patterns of involvement. Additionally, any combination of the patterns may appear.

Meningeal TB

Leptomeningitis

The most frequent manifestation of CNS TB overall and the most frequent pattern linked to complications is tubercular leptomeningitis. The organism germinates in the "rich focus," a subpial (rarely subependymal) infection focus that bursts into the subarachnoid (or ventricular) area and sets off an inflammatory reaction [7]. Although meningitis can occasionally show insidiously, particularly in youngsters, it typically manifests as meningeal irritation symptoms such as neck stiffness and photophobia [8]. The most reliable MRI finding was the diffuse enhancement of exudates within the basal cisterns. In the affected areas, diffuse leptomeningeal enhancement is visible on post-contrast T1W images [9].

Pachymeningitis

Compared to tubercular leptomeningitis, tubercular pachymeningitis is a rather rare condition. Although there have been a few reports of direct bacterial seeding of the dura through hematogenous dissemination leading to isolated pachymeningitis, the great majority of pachymeningitis cases are related to acute or chronic tubercular leptomeningitis. It is distinguished on MRI by intense post-contrast dural enhancement and focal or widespread dural thickening. The FLAIR sequence is particularly useful for visualizing thickened dura [10,11]. The diagnosis is clear in instances linked with other typical signs of CNS TB. However, in isolated cases, distinguishing this condition from other sources of pachymeningitis can be challenging, including neurosarcoidosis, autoimmune diseases (such as Wegener's granulomatosis and rheumatoid arthritis), neurosyphilis, and idiopathic hypertrophic pachymeningitis [12,13].

Parenchymal TB

Parenchymal Tuberculomas

The most common kind of intracranial parenchymal TB forms from merged tubercular microgranulomas, often at the grey-white matter junction due to hematogenous microbial arrest or via CSF spread through Virchow-Robin spaces [14]. They can occur in various brain regions, including sulcal spaces, brainstem, cerebellum, basal cisterns, and ventricles, often clustering or coalescing. More common in children (infratentorial) than adults (supratentorial), tuberculomas are granulomas with central caseous necrosis, presenting with headaches, seizures, increased intracranial pressure, focal deficits, and fever. These lesions evolve through four distinct phases: non-caseating granuloma, caseating granuloma, caseating granuloma with liquefaction, and calcified granuloma, characterized by epithelioid cells, Langhans giant cells, and surrounding lymphocytes [15,16]. During the caseating phases, T2-weighted sequences show hypointensity due to fibrosis, gliosis, and free radicals produced by macrophages [10]. Surrounding edema demonstrates hyperintensity on T2-weighted and FLAIR images, which resolves during the calcified phase when GRE/SWI sequences can identify calcifications. Imaging findings alone cannot differentiate calcified tuberculous granulomas from other etiologies. Differential diagnoses for ring-enhancing lesions include neurocysticercosis, metastases, CNS lymphoma, toxoplasmosis, glioblastoma, and pyogenic abscesses. Miliary tuberculomas, common in immunocompromised patients, arise from hematogenous spread, often pulmonary, appearing as small (<2-3 mm), diffusely scattered, non-caseating granulomas with homogeneous post-contrast enhancement [17].

Giant Tuberculomas

Giant intracranial tuberculomas, defined as lesions >2-2.5 cm, resemble tumors on MRI, complicating differentiation without clear TB evidence [18]. They demonstrate a lipid-lactate peak on MRS and reduced perfusion in central areas on magnetic resonance perfusion imaging.

Tubercular Cerebritis

Tubercular cerebritis, with or without meningitis, involves localized brain parenchyma infection, appearing as T1 hypointense, T2 hyperintense gyri with patchy contrast enhancement on MRI [19]. On pathological examination, it consists of microgranulomata with minimal bacilli and no caseous necrosis.

Tubercular Abscesses

Tubercular abscesses mimic tuberculomas as ring-enhancing lesions on MRI but lack granulomatous reaction on microscopic examination, showing a T2 hypointense rim and T2/FLAIR hyperintense pus with diffusion restriction [14]. They contain dense bacilli, progress faster, and may require surgical drainage. Differential diagnoses include pyogenic and fungal abscesses [20].

Tuberculous Rhombencephalitis

A rare neurotuberculosis form (<5% cases, ~25% in AIDS), it affects the cerebellum and brainstem, presenting as tuberculomas or leptomeningitis, or edema with T2W/FLAIR hyperintensities. Complications include cranial nerve palsies and hydrocephalus. Differential diagnoses include *Listeria* infection, herpes, Behçet’s disease, systemic lupus erythematosus (SLE), and paraneoplastic syndromes. MRS shows a lipid peak (1.3 ppm) versus elevated choline (3.2 ppm) in gliomas [21,22]. It has a poor prognosis compared to other forms of neurotuberculosis, with a high risk of mortality and significant neurological sequelae due to brainstem involvement.

Tuberculous Encephalopathy

Common in young children, it involves sudden seizures, altered mental status, stupor, or coma without meningitis signs, believed to be caused by a type IV hypersensitivity reaction [23]. MRI shows extensive T2W/FLAIR hyperintense cerebral edema and white matter damage, with a poor prognosis [24]. Acute disseminated encephalomyelitis (ADEM) is a key differential diagnosis.

Tumor-Like Appearance

Studies note intra-axial tuberculous lesions mimicking clinically and radiologically as tumors, with younger age, TB contact, or extracranial TB suggesting suspicion for non-neoplastic etiology [25,26]. Tuberculomas show hypoperfused centers (relative cerebral blood volume rCBV 0.42±0.25) and hyperperfused walls (rCBV 2.04±0.61) versus elevated rCBV in metastases [27].

Complications

CNS TB complications include hydrocephalus (most common, often communicating due to basal cistern obstruction) [28], ventriculitis (T1W hyperintense CSF, ependymal enhancement), choroid plexitis (enhanced choroid plexus) [10], and vascular issues (vasculitis in 20-41%, affecting lenticulostriate arteries, or rare dural venous sinus thrombosis) [29]. DWI is especially useful for detecting infarcts, while magnetic resonance angiography (MRA) can identify arterial irregularities, such as beading, narrowing, and segmental stenosis. Likewise, magnetic resonance venography (MRV) is useful for verifying suspected cases of cerebral venous sinus thrombosis [15]. Cranial nerve palsies (17-40%) involve nerves II, III, IV, and VII due to vasculitis or compression [16,17].

Public health awareness

This study contributes to public health by enhancing awareness and diagnostic accuracy for CNS TB, a critical and often under-recognized form of extrapulmonary TB. In regions with high TB prevalence, like India, the findings of this study can inform doctors at both tertiary and secondary care levels. This has downstream effects on national TB control programs by minimizing misdiagnosis and inappropriate treatment, especially in resource-constrained settings where delayed diagnosis often results in advanced disease with a worse prognosis.

Limitations

Our study has a few limitations. First, laboratory confirmation or histopathological analyses through surgical biopsy were not obtained in all cases; some diagnoses were presumptive, based on evidence of coexisting cranial or extracranial TB. Second, our study included a relatively limited sample size.

## Conclusions

CNS TB is a multifaceted disease with varied imaging presentations, manifesting in a broad spectrum of brain patterns, including tubercular meningitis, tuberculoma, tubercular cerebritis, abscess, and encephalopathy. Each of these patterns presents distinct appearances and can closely resemble various other conditions. A comprehensive evaluation of MRI findings, the effective use of advanced imaging techniques, and robust clinicopathological correlation allow us to achieve accurate and timely diagnoses. Strengthening diagnostic capacity at early stages aligns with the WHO’s End TB Strategy goals. The study highlights the importance of interdisciplinary collaboration among neurologists, radiologists, and pathologists.
